# Metastatic renal cell carcinoma presenting as multiple cutaneous lesions visualized through reflectance confocal microscopy

**DOI:** 10.1002/ccr3.7486

**Published:** 2023-06-07

**Authors:** Shazli Razi, Samantha Ouellette, Samavia Khan, Babar Rao

**Affiliations:** ^1^ Rao Dermatology Atlantic Highlands New Jersey USA; ^2^ Department of Pathology & Laboratory Medicine Rutgers Robert Wood Johnson Medical School, Center for Dermatology Somerset New Jersey USA

**Keywords:** cancer, dermatopathology, metastases, noninvasive imaging, reflectance confocal microscopy, skin cancer

## Abstract

We present the first case of metastatic renal cell carcinoma visualized via reflectance confocal microscopy (RCM). This case describes the RCM features of such a tumor, in an effort to improve noninvasive characterization of cutaneous metastases.

## Graphical abstract

We present a case of metastatic renal cell carcinoma is described on reflectance confocal microscopy.

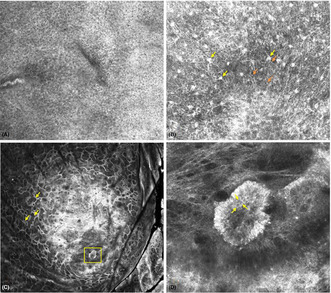

## 1 INTRODUCTION

Cutaneous metastases from internal organs, although rare, can signify a poor prognosis. In addition to malignant melanoma, cancers of lung, colon, ovary, and breast are associated with cutaneous metastasis. Renal cell carcinoma (RCC) is considered the most lethal urologic tumor.^1^ The mean age of presentation is 66 years, and it presents more commonly in men than in women (2:1).^2^ The classic triad of RCC includes flank pain, hematuria, and a palpable abdominal mass, however, it is uncommon to see concurrent presentation of all three findings in one patient.^3^

## 2 CASE REPORT

### 2.1 Clinical features

**FIGURE 1:**
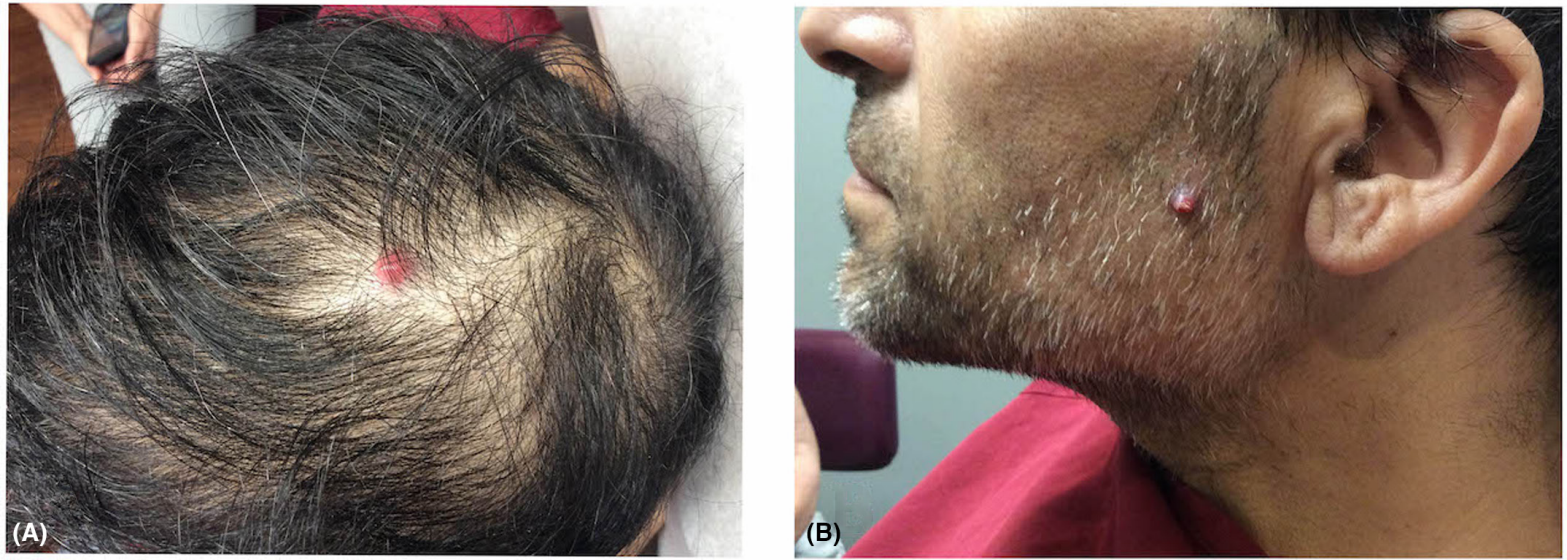
Erythematous papules on the (A) left anterior scalp and (B) left cheek.

A 47‐year‐old man presented to the dermatologist with erythematous papules on the left anterior scalp (Figure 1A) and left cheek (Figure 1B) that have been progressively enlarging for the past 3 weeks. The patient had a history of renal cell carcinoma and was undergoing radiation therapy. The patient opted for reflectance confocal microscopy before proceeding with biopsy of both cutaneous lesions.

### 2.2 Reflectance confocal microscopy characteristics

**FIGURE 2:**
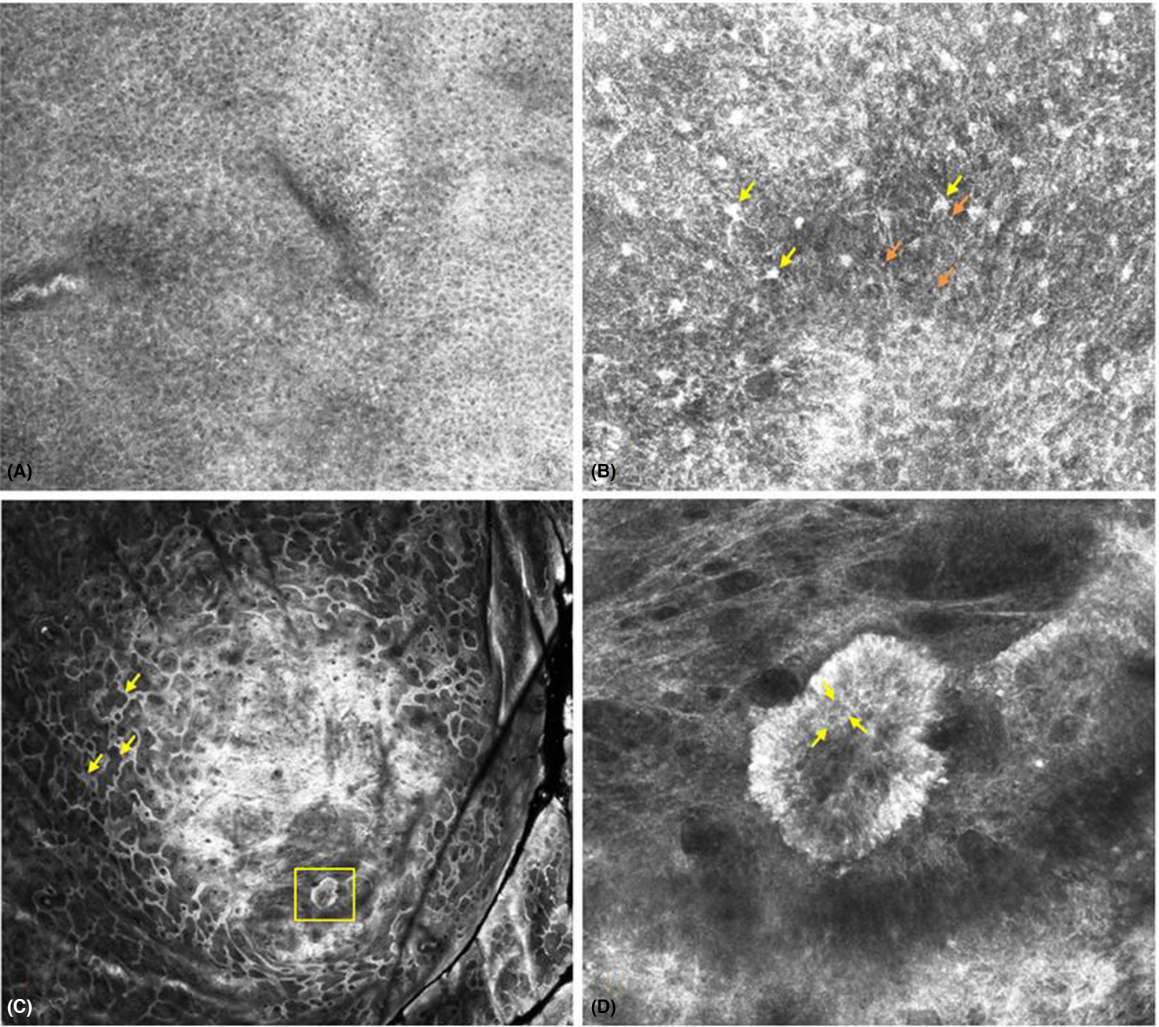
Metastatic renal cell carcinoma on reflectance confocal microscopy. (A) Honeycomb pattern in the epidermis. (B) Large, bright dendritic cells (yellow arrows) and small, bright cells in the epidermis (orange arrows). (C) A well‐defined, hyper‐refractile lesion surrounded by edged papillae at the level of the dermal–epidermal junction (yellow arrows). A lobule can be seen in the dermis (yellow box). (D) Zoomed view of the lobule consisting of polygonal, interconnected cells, likely epithelial cells distinct from surrounding keratinocytes, in the dermis (yellow arrows).

RCM images show a well‐defined, hyper‐refractile lesion at the center of the image (Figure 2). Honeycomb pattern and small bright cells and large bright dendritic cells can be seen in the epidermis. Edged papillae at the level of the dermal–epidermal junction can be seen surrounding a central, homogeneous area. A lobule of polygonal, interconnected cells, likely epithelial cells distinct from surrounding keratinocytes, can be seen in the dermis at the inferior margin of the lesion. Superior margin shows polycyclic rings representing dermal–epidermal junction, surrounding and overlying a homogenous refractile area (Figure 2C). The RCM features are suggestive of a metastatic tumor, as it lacks features of a squamous cell carcinoma, basal cell carcinoma, melanocytic tumor, and other epithelial infiltrates. Other epithelial infiltrates such as sarcoidosis, foreign body granulomas, and lymphomas can also be excluded. For example, sarcoidosis will have large macrophages which will be visualized under RCM which this case lacks. Foreign body granuloma can be excluded as there is no evidence of foamy/multinucleated giant cells surrounded by fibrous response. Lymphoma can be excluded as there is no evidence of lymphocytes that are visible as hyperreflective bright cells with high density.

### 2.3 Gross characteristics

Specimen A (left anterior scalp) was grossly oval shaped, measuring 9 × 4 mm on the surface and 2 mm deep. Specimen B (left cheek) was grossly irregular in shape and measured 6 × 4 mm on the surface and 5 mm in height.

### 2.4 Microscopic characteristics

**FIGURE 3:**
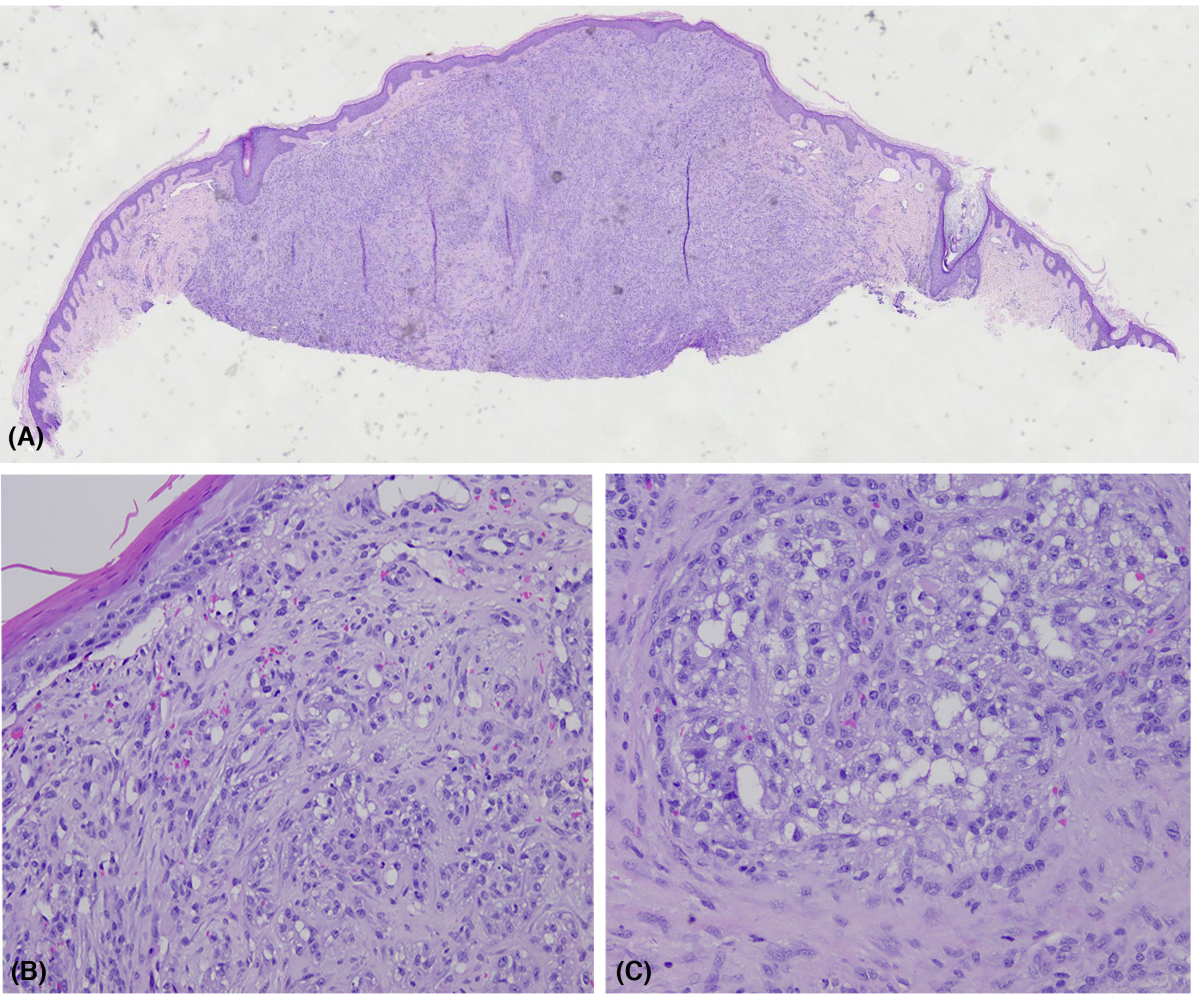
Metastatic clear cell renal carcinoma. (A) Hematoxylin–eosin ×40, (B) hematoxylin–eosin ×200, and (C) hematoxylin–eosin ×200.

**FIGURE 4:**
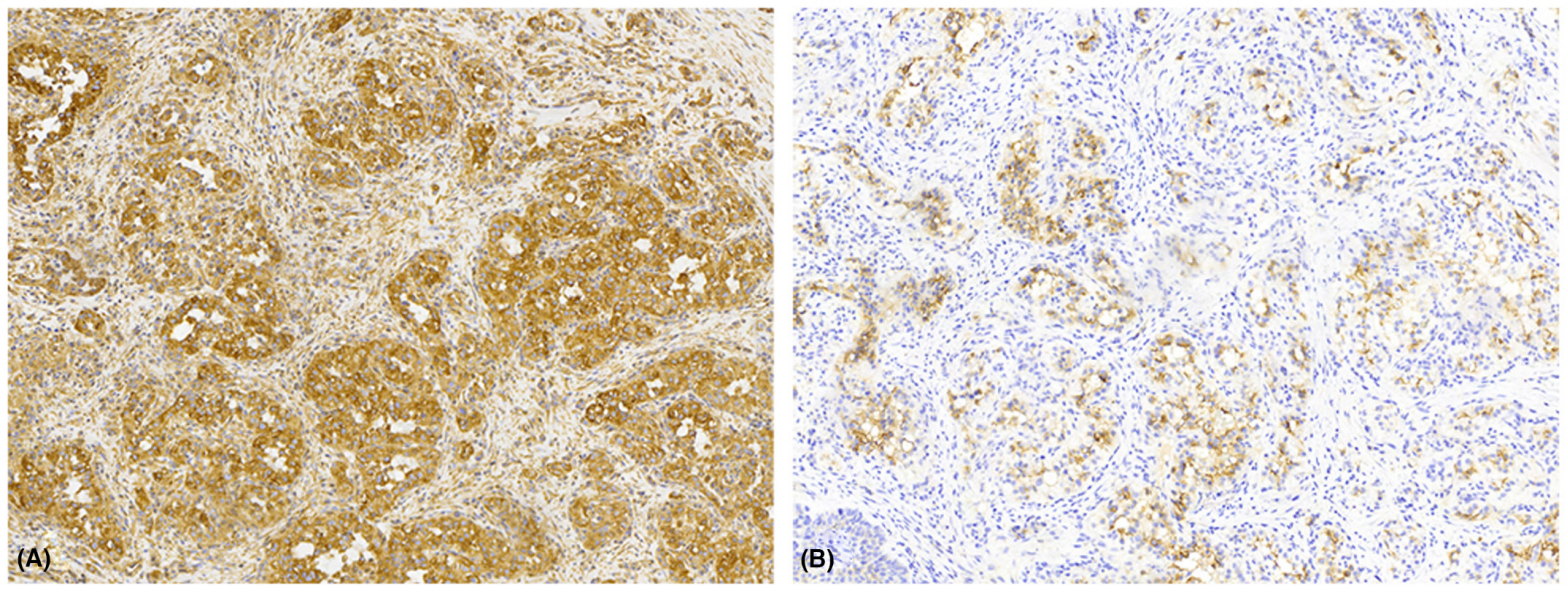
Metastatic clear cell renal carcinoma stains. (A) Vimentin positive and (B) EMA positive.

Specimens A and B present with similar microscopic findings. The lesion consisted of an intradermal nodule composed of clear cells in a glandular configuration with a prominent vascular stroma. Multiple erythrocytes were noted (Figure 3). Pancytokeratin, vimentin, and Epithelial membrane antigen (EMA) stains were positive (Figure 4). CD10 was focally positive. These findings were consistent with a diagnosis of metastatic renal cell carcinoma.

### 2.5 Correlation between histology and reflectance confocal microscopy

Histology shows irregular epidermal hyperplasia overlying a dermal nodule composed of round bluish and vacuolated cells surrounded by dermal fibrosis, telangiectasia, and chronic inflammation. RCM shows hyperplastic polycyclic loops representing dermal–epidermal junction overlying a central hyperreflective homogenous area composed of dermal fibrosis, telangiectasia, and chronic inflammation (correlating with histopathology) and a lobule in the center of the lesion (Figures 2 and 3).

## 3 DISCUSSION

Skin metastasis of renal cell carcinoma signifies advanced disease, and in the majority of cases, it may be accompanied with metastasis to other organs.^4^ Patients with cutaneous metastasis have poor prognosis.^5^ If a single lesion is present, the 5‐year survival rate is 13%–50%. In case of multiple lesions, the 5‐year survival rate decreases to 0%–8%.^6^ Most common sites for skin metastases in RCC are the scalp and face.^7^ For localized cutaneous metastasis, local excision is an option but provides little benefit.^7^ In cases of solitary skin metastasis, radiotherapy followed by chemotherapy may be beneficial.^8^ Targeted therapy for cutaneous metastases may include VEGF inhibitors (e.g., bevacizumab),^9^ tyrosine kinase inhibitors (e.g., sunitinib), or mTOR inhibitors (e.g., everolimus).^10^

Patients with a history of renal cell carcinoma who are presenting with abnormal nodular growths on their skin should be followed regularly by a dermatologist. RCM shows potential as a real‐time, noninvasive adjunct tool for assessing smooth dermal papules/nodules in the setting of suspected metastasis, melanoma, primary cutaneous malignancies, or deposition diseases. Although there are no defined RCM diagnostic features of metastasis, RCM's ability to detect atypical epidermal and dermal changes shows its potential in biopsy site selection in cases of suspected malignancy.

## AUTHOR CONTRIBUTIONS

**Shazli Razi:** Conceptualization; data curation; visualization; writing – original draft. **Samantha Ouellette:** Conceptualization; data curation; resources; writing – original draft. **Samavia Khan:** Conceptualization; writing – original draft; writing – review and editing. **Babar Rao:** Writing – original draft; writing – review and editing.

## FUNDING INFORMATION

No funding was used in this case report.

## CONFLICT OF INTEREST STATEMENT

None.

## CONSENT

Patient gave consent for photographs and medical information to be published in print and online and with the understanding that this information may be publicly available.

## Data Availability

N/A.
